# Performance and Milk Composition of Nubian Goats as Affected by Increasing Level of *Nannochloropsis oculata* Microalgae

**DOI:** 10.3390/ani10122453

**Published:** 2020-12-21

**Authors:** Ahmed E. Kholif, Gouda A. Gouda, Hatem A. Hamdon

**Affiliations:** 1 Dairy Science Department, National Research Centre, 33 Bohouth St. Dokki, Giza 12622, Egypt; gagouda@gmail.com; 2 Department of Animal Production, Faculty of Agriculture, New Valley University, New Valley 72511, Egypt; hamdon@nv.aun.edu.eg

**Keywords:** blood metabolites, digestibility coefficients, eicosapentaenoic acid, milk fatty acids, lactational performance, *Nannochloropsis oculata* microalgae

## Abstract

**Simple Summary:**

Recently, microalgae, natural marine resources, have gained increasing interests as a feed for animals. Microalgae are single-cell microorganisms that have been used to provide nutrition to humans and animals for centuries. Research has shown that inclusion of microalgae in diets improved feed utilization, milk production and quality, growth performance, and meat quality in ruminants, as a result of improved diet nutritive value leading to improved feed utilization. Very low doses of microalgae in feed enhance growth and lactational performance of ruminants. *Nannochloropsis oculata* microalgae is a rich source of rumen protected healthy fatty acids which can be explored as a feeding strategy to enhance the nutritional value of milk for consumers.

**Abstract:**

Fat supplementation affects the lactational performance of goats and dramatically changes milk nutritive value. In the present experiment, two levels of *Nannochloropsis oculata* microalgae, a natural source of rumen-protected eicosapentaenoic acid (EPA), were studied in the diet of Nubian goats. Using quintuplicated 3 × 3 Latin square design, fifteen lactating goats, (14 ± 2 months old and 33.0 ± 1.3 kg) after kidding, were randomly assigned into three treatments in an 84-d assay. Goats were offered a basal diet comprising berseem clover, wheat straw and concentrates in 3:2:5, respectively, (control treatment-no supplementation). The other two treatments were supplemented with *N. oculata* microalgae at 5 g (NOM5 treatment) or 10 g (NOM10 treatment)/doe/d. Without affecting intake, treatments improved (*p* < 0.01) nutrient digestibility. Supplementations had no effect on ruminal pH and ammonia-nitrogen, however, NOM5 and NOM10 linearly improved (*p* < 0.05) total volatile fatty acids and propionic acids. *N. oculata* supplementation linearly increased (*p* < 0.01) milk yield and lactose content. Supplementation reduced atherogenic index (*p* = 0.004) and enhanced the concentrations of unsaturated fatty acids and C20:5n3 (EPA). Conclusively, feeding Nubian goats on diet supplemented with *N. oculata* at 5 and 10 g improved milk production and the nutritive value. No improvements in the performance were observed when *N. oculata* dose was increased from 5 g to 10 g/doe; thus, 5 g dose is recommended for use.

## 1. Introduction

Microalgae are characterized with an extreme rapid growth rate [1], where a large production can be produced in marginal or non-arable land, making their use as feeds to improve food security applicable [2,3]. The main disadvantage is the high production cost [4], making them an uncompetitive feed option [3,5]. Due to the technical development, the situation may change in the near future.

Some microalgae are rich in unsaturated fatty acids (UFA). This makes them as options to improve ruminal fermentation and feed digestion [6,7,8], with high concentrations of docosahexaenoic acid (DHA) and conjugated linoleic acid (CLA) fatty acids in milk [6,9]. Moreover, some other microalgae are richly endowed with n-3 poly UFA, such as α-linolenic acid and eicosapentaenoic acid (EPA) [3,10,11]. Including microalgae in the diet of ruminants showed promising results [3,6,7,12].

*Nannochloropsis* is a rich source of rumen-protected EPA, DHA and CLA as well as all essential amino acids required for animal feed [6,10,13]. Alves et al. [11] reported that *Nannochloropsis* is a natural dietary source of EPA for ruminants. Durmic et al. [14] reported that *Nannochloropsis* contains EPA and DHA (21.5 and 3.2% fat, respectively). Archibeque et al. [13] evaluated the nutritive value of *Nannochloropsis oculata* compared with soybean meal and steam-flaked corn, and observed excellent nutritive value as acceptable protein and mineral supplements; however, feeding trials are recommended to validate animal acceptance, digestibility and performance.

Gomaa et al. [6] reported that *N. oculata* microalgae enhanced the ruminal fermentation of feeds, degradability and decreased methane (CH_4_) releasing *in vitro*. Additionally, Wild et al. [10] reported that EPA (C20:5n-3), the main fatty acids in *Nannochloropsis*, appears to be a valuable alternative feed product. High EPA concentration has health benefits, reducing both cardiovascular disease and cancer risk [15]. Additionally, the ratio of omega-3/omega-6 is about 2.7 and can be used as a source of omega-3 fatty acids [10]. Glover et al. [16] and Vahmani et al. [17] noted dietary supplementation of lactating cows with EPA and DHA (i.e., poly UFA) rich microalgae increased milk content of poly UFA.

The Nubian goat is a dual-purpose goat that carries more flesh than other dairy breeds. It produces milk with about 3.5–8% fat [18]. Therefore, we used it as a model for lactating goats to evaluate *N. oculata* microalgae supplementation. Information on the effect of supplementing *N. oculata* microalgae to lactating animals is limited. Therefore, the current study evaluated the effect of supplementing *N. oculata* microalgae to lactating Nubian goats on feed utilization, ruminal fermentation and milk production, composition, and nutritive value. Our hypothesis was that *N. oculata* is a source of naturally ruminal protected long chain n-3 fatty acids. Moreover, *N. oculata* supplementation would alter feed digestion and fermentability resulting in improved milk production and increased concentrations of milk UFA, omega-3 and CLA.

## 2. Materials and Methods

### 2.1. Goats and Experimental Design

Does care and handling were as outlined in the Guide for the Care and Use of Agricultural Animals in Agricultural Research and Teaching (Federation of Animal Science Societies, Champaign, IL, USA) and approved by the Institutional Animal Care and Use Committee of the Faculty of Agriculture, New Valley University, New Valley, Egypt.

Fifteen multiparous lactating Nubian goats, 14 ± 2 months old and weighing 33.0 ± 1.3 kg (7 days in milk), were used in a quintuplicated 3 × 3 Latin square design experiment (3 treatments, 3 periods, 5 does per treatment (resulting in 15 replicates/treatment)). Animals were housed individually in soil-surfaced pens (1.5 m^2^/doe) with water and diets supplied *ad libitum*. Does were fed according to NRC [19] recommendations on a diet containing concentrates feed, berseem clover (*Trifolium alexandrinum*) and wheat straw (*Triticum aestivum*) at 5:3:2, respectively, (Table 1).

Goats in all treatments were offered the same basal diet without supplementation (Control treatment), supplemented with 5 g (NOM5 treatment) or 10 g (NOM10 treatment) of *N. oculata*/doe/d. To ensure that the whole dose was received, the microalgae was added to the concentrate fraction of the diets during the morning feeding. Does were fed individually two times daily (08:00 and 16:00 h) in two equal proportions. Each experimental period lasted 28 days (comprised two weeks of adjustment to the diet and two weeks of measurements and sample collection). Feeds were sampled daily, composited weekly, dried at 60 °C in a forced-air oven for 48 h [20]. After, samples were ground to pass a 1-mm screen in a mill and stored for further chemical analyses.

### 2.2. Nannochloropsis oculata Microalgae

As previously explained in Gomaa et al. [6], lyophilized *N. oculata* biomass produced using BG-II growth medium [21] was used. *N. oculata* contained 92.2% dry matter (DM), 81.1% organic matter (OM), 29.2% crude protein (CP), 11.1% carbohydrates, and 29.2% oil. Chemical and fatty acids analysis of *N. oculata* have been described in details in Gomaa et al. [6]. Fatty acids profile of *N. oculata* microalgae is shown in Table 2.

### 2.3. Digestibility and Chemical Analysis

During the last two weeks of each experimental period, a digestibility trial was conducted (i.e., 3 digestibility trials). During the measurement weeks, daily feed intake was measured (the difference between served diets and orts from the previous day). Total fecal output was collected, twice daily for each goat before feeding (2 times), and stored at −10 °C for subsequent analysis. Daily fecal sample (100 g/does) was taken and pooled within a period. Feeds, orts and feces were grounded and analyzed for DM, ash, N and ether extract according to AOAC [20] official methods. Neutral detergent fiber (NDF) and acid detergent fiber (ADF) were determined using ANKOM^200^ Fiber analyzer and expressed without residual ash. Non-structural carbohydrates (NSC), OM, cellulose and hemicellulose were calculated.

### 2.4. Ruminal Fermentation

Does were sampled individually for ruminal contents 3 h after being fed in the morning on the last day of each experimental period using a stomach tube and hand pump. The pH of the ruminal fluid was taken immediately with a pH meter (HI98127 pHep^®^ 4 pH/Temperature Tester, Hanna^®^ Instrument, Villafranca padovana PD, Italy). Rumen contents were strained through four layers of cheesecloth. Approximately 5 mL of the strained rumen liquor was preserved in 5 mL of 0.2 M HCl for the ammonia-N (NH_3_-N) analysis using AOAC [20] method. Another 0.8 mL of the strained rumen liquor was mixed with 0.2 mL of a solution containing 250 g of metaphosphoric acid/L for the total volatile fatty acids (VFA) determination by titration. The concentrations and molar proportions of individual VFAs were determined by gas-liquid chromatography (model 5890, Hewlett-Packard, Little Falls, DE, USA).

### 2.5. Blood Measurements

Does were sampled for blood 4 h after morning feeding. Approximately 10 mL of blood was obtained from the jugular vein of each doe in a clean dry tube without anticoagulants. After, samples were centrifuged at 4000× *g* for 20 min before storing in 2-mL Eppendorf tubes at −20 °C pending analysis. By using specific kits (Stanbio Laboratory, Boerne, TX, USA) and following manufacturer instructions, blood serum samples were analyzed for concentrations of total proteins, albumin, urea-N, glucose, glutamate-pyruvate transaminase (GPT), glutamate-oxaloacetate transaminase (GOT), triglycerides, high-density lipoprotein cholesterol (HDL), low-density lipoprotein cholesterol (LDL), non-esterified fatty acids (NEFA) and beta-hydroxybutyric acid (BHBA) by Ultraviolet–Visible spectrophotometry. Globulin concentration was calculated by subtracting albumin values from their corresponding total protein values.

### 2.6. Lactational Performance

Does were hand milked twice daily at 09:00 and 21:00 h during the last two weeks of each experimental period. Both the morning and evening milk were mixed together and 10% of the recorded milk yield was taken daily and analyzed for different components (total solids, solids not fat, fat, protein, lactose and ash) using infrared spectrophotometry (Milkotester LM2, Belovo, Bulgaria).

Milk was analyzed for fatty acid proportions using methyl esters prepared by base-catalyzed methanolysis of the glycerides (NaOH in methanol), following the standards of the International Dairy Federation. Samples were analyzed using an Agilent 19091J-413 HP-5 column containing 5% phenyl methyl siloxane (30 m × 0.32 mm i.d., df = 0.25 μm; Agilent Technologies Inc., Palo Alto, CA, USA) on a gas chromatography (Hewlett-Packard, Model 6890, Palo Alto, CA, USA) equipped with a flame ionization detector.

Milk gross energy, fat-corrected milk (FCM) and energy-corrected milk (ECM) were estimated according to Tyrrell and Reid [22], NRC [23] and Sjaunja et al. [24] equations, respectively.

### 2.7. Statistical Analyses

The Shapiro-Wilk test was used to test the normal distribution of variables. For the small number of variables that showed significance for the Shapiro-Wilk test, data transformation (e.g., natural log, inverse of the natural log, square root, and inverse of the square root) was applied before reanalyzing the normality of the residuals. Using a quintuplicate 3 × 3 Latin square design with three periods and three treatments, data were analyzed with PROC MIXED of SAS 9.4 (SAS Inst., Inc., Cary, NC, USA). Individual does were the experimental units. The statistical model was: Y_ijkl_ = μ + S_i_ + A_j_ + T_k_ + D_l_(S_i_) + E_ijkl_, where Y_ijkl_ is each individual observation for a given variable, μ is the overall mean, S_i_ is the square effect, A_j_ is the treatment effect, T_k_ is the period effect, D_l_(S_i_) is the effect of a doe within the square and E_ijkl_ is the residual error. When *F*-test was significant at *p* < 0.05, values of means were compared using the difference probability option of the least squares mean statement. Polynomial (linear and quadratic) contrasts were used to describe responses to increasing doses of *N. oculata*.

## 3. Results

### 3.1. Growth, Milk Yield, Composition, Efficiency and Fatty Acid Profile

For initial and final body weight as well as daily weight changes, no differences were observed with feeding NOM5 and NOM10 treatments (Table 3). Both of NOM5 and NOM10 treatments linearly improved (*p* < 0.01) daily milk production expressed as actual, ECM and FCM, as well as the yields of milk components (*p* < 0.05). Additionally, NOM5 and NOM10 enhanced the lactose concentration relative to the control. Linear increase in feed efficiency expressed as actual milk production/feed consumed (*p* = 0.019) or ECM/feed consumed (*p* = 0.035) were observed with NOM5 and NOM10 treatments (Table 3).

As shown in Table 4, without affecting milk individual fatty acids or total SFA concentrations, NOM5 and NOM10 treatments reduced (*p* = 0.002) C16:0 concentration and atherogenic index (*p* = 0.004) but improved (*p* < 0.01) of C20:5n3 (EPA), UFA and mono UFA concentrations in relation to the control treatment.

### 3.2. Voluntary Intake and Nutrient Digestibility

*N. oculata* supplementation had no effect on feed consumption (Table 5). Except for EE digestibility, NOM5 and NOM10 supplementations enhanced (*p* < 0.01) nutrient digestibility. Relative to the control treatment, NOM5 and NOM10 treatments showed higher (*p* < 0.001) digestible nutrients and energy value.

### 3.3. Serum Metabolites and Rumen Fermentation

Treatments had no effect serum total protein, albumin, globulin and urea-N (Table 6). Moreover, NOM5 and NOM10 had no effect on serum GOT and GPT, triglycerides, LDL, NEFA, HDL or BHBA concentrations; however, they increased glucose concentration.

Dietary treatments had no effect ruminal pH or ammonia-N (Table 7). *N. oculata* linearly increased total VFA (*p* = 0.002) and propionic acid (*p* = 0.022) without affecting ruminal acetic and butyric acid proportions. Moreover, the NOM10 treatment linearly decreased (*p* = 0.045) calculated CH_4_ production.

## 4. Discussion

### 4.1. Lactational Performance

The NOM5 and NOM10 linearly increased daily milk yield for the actual (by about 10.6 and 1.6%), ECM (by 10.2 and14.1%) and FCM (by 10 and 13.1%) respectively. Increased milk yield with unaffected feed consumption results in enhanced feed (milk) efficiency by about expressed as actual milk production/feed intake (by 9.5 and 13.4%, respectively) or ECM/feed intake (by 9.1 and 12.8%, respectively). Enhanced nutrient digestion and ruminal fermentation are majorly responsible for the enhanced milk yield [27]. As it was discussed later, increased lactose content with *N. oculata* may also be responsible for the improved milk yield [28].

*N. oculata* increase the concentration of lactose (by 5 and 6.3% for NOM5 and NOM10 treatments respectively) as a result of increased propionic acid production. Propionic acid, the precursor for gluconeogenesis and lactose synthesis, favorably affect milk production [28]. The minimal effect of *N. oculata* on milk fat concentration is in line with the result of milk fat precursors in rumen, namely acetate and butyrate as well as precursors in blood namely NEFA and BHBA [29,30]. Lamminen et al. [3] reported higher milk fat with microalgae supplementation. The level of supplementation may be responsible about the inconsistency. Póti et al. [31] observed that feeding microalga as fat supplement to Hungarian native goat increased milk fat content without affecting other milk components.

### 4.2. Milk Fatty Acid Profile

Minor changes in milk fatty acid profile were expected since microalgae supplementation did not affect fat concentration in milk; however, some changes were noted. Composition of milk fatty acid is influenced by the type of fatty acids consumed by animal. Generally, dietary factors and absorbed fats affect milk fatty acid profile [32]. With exception of C16:0 and C18:1 n9T, feeding *N. oculata* had no effect on individual fatty acids. In their review, Altomonte et al. [33] summarized that greatest changes in the profile of milk fatty acids were related to increases in long-chain PUFA and omega-3 fatty acids and to accompanying decreases in SFA.

*N. oculata* decreased and increased C16:0 and C18:1 concentrations respectively, indicating enhanced milk nutritive value for human health [34]. Vahmani et al. [17] observed a reduced C16:0 concentration by 12% with PUFA-rich microalgae to lactating cows. This may be due to the suppression of mammary *de novo* fatty acids synthesis [35]. Moreover, increased C18:1 fatty acid relates to increased mammary supply of C18:0 with microalgae supplementation, providing more substrate for mammary Δ^9^-desaturase.

*N. oculata* fed goats produced EPA fatty acid-fortified milk (increases of 12.8 and 36.1% respectively for NOM5 and NOM10 treatments), without affecting DHA concentration. This may be a result of the inclusion of EPA-rich microalgae in the diet [3]. Efficiencies of transfer of both of EPA and DHA from diet to milk have been estimated to be 2.6 and 4.1%, respectively [36] and can be increased several fold when animals’ diet is supplemented with sources protected from ruminal biohydrogenation [37,38]. Lamminen et al. [3] reported increased EPA concentration fourfold with *N. oculata* feeding to lactating cows. Moreover, Vahmani et al. [17] observed dietary supplementation of lactating cows with EPA-rich supplementation improved milk fat concentrations of EPA by 100% and DHA by 86%.

Theoretically, majority of consumed poly UFA undergo ruminal biohydrogenation and are thus not incorporated into milk unaltered; however, protecting them from ruminal biohydrogenation increases their excretion in milk. *N. oculata* increased UFA by 6.3 and 11.2%, respectively for NOM5 and NOM10 treatments, resulting in production healthier milk for human consumption. Vahmani et al. [17] observed a decreased SFA by 9% and increased C18:1 n9T concentration by 81% with UFA-rich microalgae. Boeckaert et al. [39] reported that UFA-rich microalgae suppress *in vitro* biohydrogenation of fatty acids, resulting in reduced SFA and increased UFA.

The gradual increases in C20:5n-3 (α-linolenic acid) proportions with increasing *N. oculata* indicates improve nutritive value of produced milk. A raise in EPA has been reported in cows supplemented with full-fatted microalgae biomass [40]. This result indicates that microalgae biomass may play a positive role in enhancing the health-promoting n-3 fatty acids in milk.

*N. oculata* decreased the concentrations of atherogenic index by 9.3 and 16.1% respectively for NOM5 and NOM10 treatments, buttressing the fact that feeding microalgae produced healthy milk. With the supplementation of rich fat microalga to Hungarian native goat, Póti et al. [31] noted increases in the concentrations of C4:0, C18:1t, C18:2, C18:3, C20:3, C20:5, C22:6, mon UFA, poly UFA, and n-3 fatty acids and decreases in the concentrations of C20:4 and SFA in milk. Additionally, they [31] observed that increasing the supply of n-3 poly UFA decreased the ratios of n-6/n-3 fatty acids and the atherogenic index.

### 4.3. Intake and Digestibility

Unexpectedly, *N. oculata* had no effect on feed consumption indicating unaffected palatability of the diets by the microalgae supplementation. Glover et al. [16] noted that feed consumption was not affected with feeding of ruminally-protected microalgae rich in DHA fatty acids. Feeding large quantities of microalgae causes palatability problems in ruminants as a result odor and taste of the microalgae [3] due to the ‘grassy, vegetable, cucumber’ flavors [41]. Lamminen et al. [3] observed minimal effects with feeding microalgae (*Spirulina, Chlorella* and *Nannochloropsis*) in the concentrate portion of a diet to lactating cows; however, they noted increased silage intake and decreased concentrate intake. The level of microalgae supplementation may be a reason for the inconsistency between experiments. In the present experiment, microalgae supplementation ranged between 0.49 to 0.94% compared with about 10-fold supplementation in Lamminen et al. [3].

*N. oculata*, at the two levels evaluated in the present experiment, enhanced nutrient digestibility without affecting the digestibility of EE, revealing enhanced ruminal fermentation with the microalgae supplementation. The microalgae contains about 67% UFA of its content of total fatty acids which was expected to negatively affect fiber digestion [42,43] due to the adverse effect of UFA on the rumen microbiome [42,43]. These results reveal that the two rates/doses of microalgae in the current assay fell within tolerable range, as previously noted with feed intake. Additionally, result of digestibility confirms the result of Alves et al. [11] who reported that the UFA in *N. oculata* are in a protected form, which means that minimal ruminal biohydrogenation occurs to microalgae UFA and prevents their negative effects on ruminal microbiota. Otherwise, the increased nutrient digestibility indicates improved rumen microflora population/activity with the supplementation. Supplementation with UFA decreases the number of protozoa which causes increased total bacterial population as no or reduced predation of bacteria by protozoa occurs [6]. This result concurs with those of Gomaa et al. [6] who observed enhanced DM and NDF digestibility with *N. oculata* and sunflower oil mixture due to improved ruminal microbes’ activity.

### 4.4. Serum Metabolites

All serum parameters in the present study were within the recommended ranges for healthy animals [44]. *N. oculata* supplementation did not affect blood total protein, protein fractions and urea-N, indicating marginal effects on the nutritional status and muscles protein catabolism [44]. Moreover, these results suggest normal activity. Serum urea-N is a good index of the kidney glomerular filtration [45]. Moreover, *N. oculata* did not affect the concentrations of serum GOT and GPT indicating minimal effects on liver function and reflects a high safety of feeding *N. oculata* to goats.

*N. oculata* did not affect the concentrations of serum triglycerides, LDL and HDL, indicating unaffected release of triglyceride-rich lipoproteins into the lymphatic system. Additionally, *N. oculata* did not affect the concentrations of serum NEFA and BHBA, revealing positive effects on minimizing body fat break down in goats supplemented with *N. oculata*. Such results suggest absence of a negative energy balance in the goats. Moreover, this indicates enhanced energy status of goats with *N. oculata* supplementation. These results are in line with the results of unaffected daily body weight loss.

*N. oculata* increased serum glucose concentration with 10.8 and 12.2%, for NOM5 and NOM10 treatments, respectively which is the consequence of improved OM digestibility, total VFA concentration and propionic acid production, because about 73% of hepatic glucose production in ruminants is from ruminal propionic acid [46]. Interestingly, serum glucose results were paralleled with those of milk production, confirming the previously noted positive correlation between serum glucose and milk production in animals fed UFA supplemented diet [47].

### 4.5. Ruminal Fermentation

Values of ruminal pH [48] and concentrations of ruminal NH_3_-N [49] fell within the accepted ranges required for optimal ruminal microflora growth and activity, with no effect due to *N. oculata* feeding. Ruminal NH_3_-N concentration is an indirect evidence of unaffected duodenal flow of microbial N and efficiency [43]. That ruminal NH_3_-N was not affected, and digestibility of CP was improved with *N. oculata* feeding is an evidence of increased ruminal microbial synthesis and activity due to use of ruminal NH_3_-N and conversion of N into microbial protein. Kholif et al. [50] observed no alterations in rumen ammonia-N with UFA supplementation to lactating goats.

*N. oculata* improved ruminal total VFA concentration (by 8.1 and 15.5% for NOM5 and NOM10 treatments respectively), without affecting ruminal pH possibly due to the enhanced OM and fibers digestibilities [51]. Feed digestion and activity of ruminal microflora determine the concentration of ruminal VFA [52]. Kholif et al. [50] showed that supplementing lactating goats with ruminally protected UFA (i.e., flaxseed seeds) increased ruminal VFA concentration through the reduction of ruminal protozoa, and increasing the dietary energy density with supplementation [53].

*N. oculata* increased the ruminal propionic acid proportions by 9.4 and 11.1% respectively for NOM5 and NOM10 treatments, reflecting a higher conversion of glycerol into propionic acid [49]; glycerol is the consequence of the breakdown of dietary fatty acids. Increased propionate concentration is due to improved OM and NSC digestibility. Moreover, it may also due to diversion of excess decreased NADH to propionic acid production as a result of improved accumulation of H due to inhibition of ruminal methanogens [54].

*N. oculata* did not affect ruminal acetic and butyric acids proportions, which is another evidence that the amounts of UFA from the microalgae in the present experiment were adequate to improve the goats’ productive performance with no effect on ruminal cellulolytic bacterial activity.

*N. oculata* at high level (i.e., NOM10 treatment) decreased calculated CH_4_ production by 10.1%. Wild et al. [55] reported that *N. oculata* decreased ruminal CH_4_ production *in vitro*. Additionally, Boeckaert et al. [56] and Fievez et al. [57] observed a declined ruminal methanogenesis *in vitro*. The presence of EPA and DHA fatty is the main reason [55,56,57]. Wild et al. [55] reported a negative relationship between CH_4_ and the EPA concentration (r = −0.51). However we did not measure ruminal microbiota, assumption that the presence of UFA in the algae can be toxic to ruminal protozoa, which is one of CH_4_ producers [58]. Protozoa are the main engulfers of rumen bacteria cells (20,000 cells/h) [59]. Boeckaert et al. [39] reported that microalgae rich in UFA decreased the number of *Isotricha prostoma* and *Isotricha intestinalis* and some species of *Epidinium caudatum* ciliates.

## 5. Conclusions

Supplementation lactating Nubian goats’ diet with *N. oculata* at 5 or 10 g/doe daily enhanced daily milk production and profile of milk fatty acids as increasing UFA and C20:5n-3 (α-linolenic acid) and decreasing SFA concentrations. Moreover, *N. oculata* enhanced nutrient digestion, and ruminal fermentation without affecting blood chemistry. The 5 g/d of *N. oculata* microalgae is suggested for its using in lactating Nubian goats due to the cost.

## Figures and Tables

**Table 1:** Chemical composition of experimental control diet fed to the Nubian goats (g/kg DM basis unless otherwise stated).

	Concentrate Feed Mixture ^1^	Berseem Clover	Wheat Straw	Control (Basal) Diet ^2^
Dry matter (g/kg wet material)	907	214	904	699
Organic matter	895	858	912	887
Crude protein	154	144	37	128
Ether extract	44.0	49.9	10.7	39.1
Non-structural carbohydrate	448	213	116	311
Neutral detergent fiber	249	451	748	409
Acid detergent fiber	140	348	505	275
Cellulose	115	296	368	220
Hemicellulose	109	104	243	134

^1^ Consisted of 55% corn, 25% wheat bran, 17% soybean meal, 2% limestone, 0.5% NaCl and 0.5% mixture of minerals and vitamins. ^2^ Control basal diet comprising (/kg DM): 500 g of concentrates feed, 300 g berseem clover and 200 g wheat straw.

**Table 2:** Fatty acids profile of *N. oculata* microalgae.

Item	g/kg Total Fatty Acids
C12:0	3.1
C14:0	72.2
C15:0	12
C16:0	205
C16:1	202
C16:2	104
C17:0	11
C18:0	22
C18:1	33
C18:2 (omega-6)	34
C18:3 (omega-3)	11
C20:5n-3	283
C22:0	4.1
Total saturated fatty acids (SFA)	329
Total monounsaturated fatty acids	236
Total polyunsaturated fatty acids	432
Total unsaturated fatty acids (UFA)	667
UFA/SFA	2.0

**Table 3:** Weight changes, milk production and composition of lactating Nubian goats fed a basal diet supplemented with *N. oculata*.

	Treatments ^1^			SEM	*p* Value			
	Control	NOM5	NOM10		Treatment	Period	Linear	Quadratic
Body weight, kg								
Initial	32.9	33.2	32.9	0.31	0.797	0.725	0.904	0.510
Final	31.6	31.9	31.8	0.47	0.896	0.024	0.789	0.702
Daily changes, g/d	−16.0	−14.9	−13.2	5.55	0.938	0.063	0.725	0.959
Production, g/d								
Milk	1034 ^b^	1144 ^a^	1185 ^a^	30.7	0.004	0.091	0.001	0.368
ECM	973 ^b^	1073 ^a^	1110 ^a^	29.4	0.006	0.042	0.002	0.392
FCM (4%)	989 ^b^	1088 ^a^	1118 ^a^	29.5	0.010	0.045	0.004	0.348
Total solids	126 ^b^	140 ^a^	145 ^a^	3.8	0.003	0.064	0.008	0.365
Solids not fat	87.4 ^b^	97.8 ^a^	102 ^a^	2.7	0.001	0.095	0.003	0.384
Protein	34.0	36.0	37.6	1.06	0.065	0.018	0.021	0.875
Fat	38.3 ^b^	42.0 ^a^	42.9 ^a^	1.19	0.023	0.032	0.009	0.351
Lactose	45.0 ^b^	52.4 ^a^	54.9 ^a^	1.48	<0.001	0.283	<0.001	0.198
Ash	8.45 ^b^	9.46 ^a^	9.84 ^a^	0.28	0.003	0.158	0.001	0.365
Milk energy content, MJ/kg	3.01 ^b^	3.33 ^a^	3.45 ^a^	0.091	0.005	0.046	0.002	0.362
Milk composition, g/kg								
Total solids	122	122	123	0.9	0.818	0.765	0.529	0.993
Solids not fat	84.6	85.4	86.4	0.65	0.178	0.990	0.065	0.931
Protein	32.9	31.4	31.7	0.41	0.308	0.186	0.059	0.075
Fat	37.1	36.7	36.2	0.43	0.288	0.249	0.119	0.879
Lactose	43.6 ^b^	45.8 ^a^	46.4 ^a^	0.45	0.003	0.280	0.001	0.157
Ash	8.18	8.28	8.30	0.102	0.673	0.052	0.409	0.750
Milk energy output, MJ/d	2.92	2.91	2.91	0.024	0.979	0.431	0.861	0.919
Milk efficiency								
Milk/DMI	0.98 ^b^	1.07 ^a^	1.11 ^a^	0.032	0.019	0.061	0.006	0.501
ECM/DMI	0.92 ^b^	1.00 ^a^	1.04 ^a^	0.031	0.033	0.035	0.011	0.519

Means with different superscripts within a row differ (*p* < 0.05). DMI, dry matter intake; ECM, energy corrected milk; FCM, fat corrected milk; SEM, standard error of the mean. ^1^ Control diet comprising (/kg DM): 500 g of concentrates feed, 300 g berseem clover and 200 g wheat straw without supplements (Control treatment) or with 5 g (NOM5 treatment) or 10 g of *N. oculata*/doe/d (NOM10 treatment) supplement.

**Table 4:** Fatty acids profile (g/100 g total fatty acids) in milk of lactating Nubian goats fed a basal diet supplemented with *N. oculata*.

	Treatments ^1^			SEM	*p* Value			
	Control	NOM5	NOM10		Treatment	Period	Linear	Quadratic
C4:0	2.99	3.00	2.63	0.312	0.630	0.803	0.412	0.626
C6:0	2.11	1.94	2.02	0.133	0.681	0.040	0.632	0.468
C8:0	2.24	2.23	2.22	0.116	0.991	0.974	0.894	0.988
C10:0	5.24	5.18	5.14	0.174	0.915	0.475	0.679	0.965
C11:0	0.90	0.85	0.87	0.038	0.619	0.460	0.528	0.459
C12:0	3.30	3.27	3.17	0.128	0.753	0.802	0.483	0.801
C14:0	9.38	9.30	8.84	0.210	0.173	0.494	0.083	0.487
C14:1	0.68	0.67	0.68	0.082	0.989	0.844	0.993	0.883
C15:0	0.55	0.53	0.56	0.035	0.875	0.353	0.965	0.610
C16:0	26.2 ^a^	24.4 ^b^	24.3 ^b^	0.38	0.002	0.253	0.002	0.089
C16:1	1.13	1.28	1.19	0.109	0.617	0.617	0.674	0.379
C17:0	0.84	0.86	0.81	0.066	0.844	0.230	0.751	0.630
C18:0	16.3	16.7	16.3	0.30	0.631	0.551	0.971	0.343
C18:1 n9T	23.8 ^b^	25.2 ^ab^	26.9 ^a^	0.58	0.004	0.632	0.009	0.861
C18:1 n9C	2.61	2.82	2.58	0.151	0.496	0.496	0.922	0.243
trans-10, cis-12C18:2	0.27	0.29	0.30	0.015	0.474	0.562	0.227	0.975
cis-9, trans-11C18:2	0.17	0.18	0.19	0.011	0.598	0.250	0.340	0.751
C18:3 n-3	0.16	0.17	0.17	0.008	0.713	0.186	0.480	0.683
C18:3 n-6	0.38	0.39	0.40	0.016	0.616	0.046	0.337	0.867
C20:0	0.67	0.67	0.66	0.028	0.926	0.667	0.700	0.975
C20:5n-3	0.15 ^c^	0.17 ^b^	0.20 ^a^	0.009	0.002	0.132	0.006	0.510
C22:5n-3	0.21	0.22	0.21	0.011	0.804	0.587	0.716	0.586
SFA	70.8	68.9	67.5	1.58	0.303	0.650	0.116	0.775
UFA	29.6 ^b^	31.4 ^a^	32.9 ^a^	0.58	0.002	0.667	0.006	0.776
Mono UFA	28.2 ^b^	30.0 ^a^	31.4 ^a^	0.57	0.003	0.629	0.007	0.777
Poly UFA	1.35 ^b^	1.42 ^ab^	1.48 ^a^	0.035	0.049	0.368	0.015	0.908
Total CLA	0.44	0.47	0.49	0.047	0.059	0.035	0.153	0.057
Omega-6/omega-3	2.32	2.28	2.34	0.101	0.896	0.515	0.847	0.673
UFA/SFA	0.42 ^b^	0.46 ^a^	0.49 ^a^	0.012	0.003	0.659	0.007	0.827
Atherogenic index ^2^	2.28 ^a^	2.10 ^b^	1.92 ^b^	0.068	0.004	0.912	0.001	0.730

Means with different superscripts within a row differ (*p* < 0.05). CLA, conjugated linoleic acid (trans-10, cis-12 C18:2 and cis-9, trans-11 C18:2); SEM, standard error of the mean; SFA, total saturated fatty acids; UFA, total unsaturated fatty acids. ^1^ Control diet comprising (/kg DM): 500 g of concentrates feed, 300 g berseem clover and 200 g wheat straw without supplement (Control treatment) or with 5 g (NOM5 treatment) or 10 g of *N. oculata*/doe/d (NOM10 treatment) supplement. ^2^ Calculated according to Ulbricht and Southgate [25]: atherogenic index = (C12:0 + 4 × C14:0 + C16:0)/∑ of UFA.

**Table 5:** Intake and nutrient digestibility of basal diet supplemented with *N. oculata*.

	Treatments ^1^			SEM	*p* Value			
	Control	NOM5	NOM10		Treatment	Period	Linear	Quadratic
Intake, g/d	1067	1075	1072	17.0	0.944	0.282	0.837	0.788
Digestibility, g/kg								
Dry matter	581 ^b^	623 ^a^	627 ^a^	5.3	<0.001	0.019	<0.001	<0.001
Organic matter	574 ^b^	608 ^a^	610 ^a^	1.8	<0.001	<0.001	<0.001	<0.001
Crude protein	579 ^b^	615 ^a^	621 ^a^	5.8	<0.001	<0.001	<0.001	0.043
Ether extract	591	607	605	5.4	0.078	<0.001	0.067	0.175
Non-structural carbohydrates	549 ^b^	603 ^a^	613 ^a^	5.7	<0.001	0.152	<0.001	0.003
Neutral detergent fiber	555 ^b^	592 ^a^	604 ^a^	6.9	<0.001	0.002	<0.001	0.170
Acid detergent fiber	542 ^b^	583 ^a^	584 ^a^	7.5	0.003	0.151	0.004	0.039
Cellulose	551 ^b^	589 ^a^	597 ^a^	8.9	0.002	0.114	0.009	0.187
Hemicellulose	548 ^b^	592 ^a^	587 ^a^	8.3	0.001	0.126	0.002	0.023
Digestible nutrients and energy value ^2^								
Digestible crude protein, g/kg DM	535 ^b^	572 ^a^	575 ^a^	4.0	<0.001	0.448	<0.001	0.009
Digestible crude protein, g/kg DM	73.8 ^b^	78.4 ^a^	79.2 ^a^	0.74	<0.001	<0.001	<0.001	0.043
Digestible energy, MJ/kg DM	2.36 ^b^	2.52 ^a^	2.54 ^a^	0.017	<0.001	0.484	<0.001	0.008
Metabolizable energy, MJ/kg DM	2.38 ^b^	2.55 ^a^	2.56 ^a^	0.018	<0.001	0.457	<0.001	0.009
Net energy for lactation, MJ/kg DM	1.19 ^b^	1.28 ^a^	1.29 ^a^	0.010	<0.001	0.419	<0.001	0.001

Means with different superscripts within a row differ (*p* < 0.05). SEM, standard error of the mean. ^1^ Control diet comprising (/kg DM): 500 g of concentrates feed, 300 g berseem clover and 200 g wheat straw without supplement (Control treatment) or with 5 g (NOM5 treatment) or 10 g of *N. oculata*/doe/d (NOM10 treatment) supplement. ^2^ Calculated according to NRC [23].

**Table 6:** Serum metabolites (g/dL, unless stated otherwise) of lactating Nubian goats fed a basal diet supplemented with *N. oculata*.

	Treatments ^1^			SEM	*p* Value			
	Control	NOM5	NOM10		Treatment	Period	Linear	Quadratic
Total proteins	6.59	6.55	6.64	0.059	0.543	0.112	0.570	0.345
Albumin	3.43	3.47	3.46	0.035	0.713	0.391	0.597	0.531
Globulin	3.16	3.08	3.18	0.056	0.360	0.015	0.787	0.163
Albumin/globulin ratio	1.09	1.13	1.09	0.024	0.372	0.014	0.921	0.163
Urea-N	39.4	39.5	39.6	0.85	0.986	0.002	0.869	0.991
Glucose	61.9 ^b^	68.5 ^a^	68.2 ^a^	0.75	<0.001	0.188	<0.001	0.005
GPT, Units/L	15.6	15.4	15.5	0.27	0.843	0.977	0.776	0.612
GOT, Units/L	31.2	31.7	31.2	0.62	0.786	0.133	0.938	0.493
Triglycerides	140	137	134	1.9	0.093	0.546	0.030	0.919
HDL	50.4	49.7	50.1	1.01	0.897	0.758	0.817	0.688
LDL	53.7	51.8	51.4	1.60	0.570	0.918	0.323	0.711
NEFA	0.95	1.04	1.02	0.030	0.139	0.278	0.161	0.155
BHBA	1.68	1.63	1.63	0.042	0.647	0.169	0.415	0.655

Means with different superscripts within a row differ (*p* < 0.05). SEM, standard error of the mean. BHBA, Beta-hydroxybutyric acid; GOT, glutamate-oxaloacetate transaminase; GPT, glutamate-pyruvate transaminase; HDL, High-density lipoprotein cholesterol; LDL, Low-density lipoprotein cholesterol; NEFA, Non-esterified fatty acids. ^1^ Control diet comprising (/kg DM): 500 g of concentrates feed, 300 g berseem clover and 200 g wheat straw without supplement (Control treatment) or with 5 g (NOM5 treatment) or 10 g of *N. oculata*/doe/d (NOM10 treatment) supplement.

**Table 7:** Ruminal fermentation in lactating Nubian goats fed a basal diet supplemented with *N. oculata*.

	Treatments ^1^			SEM	*p* Value			
	Control	NOM5	NOM10		Treatment	Period	Linear	Quadratic
pH	5.94	6.03	6.14	0.060	0.078	0.077	0.025	0.904
Ammonia-N, g/L	25.3	26.3	27.3	0.79	0.239	0.357	0.093	0.973
Volatile fatty acids, mmol/L	115 ^c^	124 ^b^	133 ^a^	2.700	0.002	<0.001	<0.001	0.901
Acetic, mmol/100 mmol	56.5	57.4	55.1	1.63	0.617	0.799	0.565	0.430
Propionic, mmol/100 mmol	25.0 ^b^	27.4 ^a^	27.8 ^a^	0.850	0.045	0.468	0.031	0.356
Butyric, mmol/100 mmol	16.4	13.5	13.5	1.29	0.201	0.381	0.120	0.372
Acetic/propionic ratio	2.31	2.11	2.00	0.123	0.223	0.413	0.090	0.799
Methane production^2^	25.1 ^a^	23.7 ^ab^	22.5 ^b^	0.670	0.045	0.943	0.014	0.882

Means with different superscripts within a row differ (*p* < 0.05). SEM, standard error of the mean. ^1^ Control diet comprising (/kg DM): 500 g of concentrates mixture, 300 g berseem clover and 200 g wheat straw without supplement (Control treatment) or with 5 g (NOM5 treatment) or 10 g of *N. oculata*/doe/d (NOM10 treatment) supplement. ^2^ Methane production (mmol/L) = 0.45 (Acetic) − 0.275 (Propionic) + 0.4 (Butyric) [26].
